# Cumulative Number of Cell Divisions as a Meaningful Timescale for Adaptive Laboratory Evolution of *Escherichia coli*

**DOI:** 10.1371/journal.pone.0026172

**Published:** 2011-10-18

**Authors:** Dae-Hee Lee, Adam M. Feist, Christian L. Barrett, Bernhard Ø. Palsson

**Affiliations:** Department of Bioengineering, University of California San Diego, La Jolla, California, United States of America; Texas A&M University, United States of America

## Abstract

Adaptive laboratory evolution (ALE) under controlled conditions has become a valuable approach for the study of the genetic and biochemical basis for microbial adaptation under a given selection pressure. Conventionally, the timescale in ALE experiments has been set in terms of number of generations. As mutations are believed to occur primarily during cell division in growing cultures, the cumulative number of cell divisions (CCD) would be an alternative way to set the timescale for ALE. Here we show that in short-term ALE (up to 40–50 days), *Escherichia coli*, under growth rate selection pressure, was found to undergo approximately 10^11.2^ total cumulative cell divisions in the population to produce a new stable growth phenotype that results from 2 to 8 mutations. Continuous exposure to a low level of the mutagen *N*-methyl-*N*′-nitro-*N*-nitrosoguanidine was found to accelerate this timescale and led to a superior growth rate phenotype with a much larger number of mutations as determined with whole-genome sequencing. These results would be useful for the fundamental kinetics of the ALE process in designing ALE experiments and provide a basis for its quantitative description.

## Introduction

**Figure 1:**
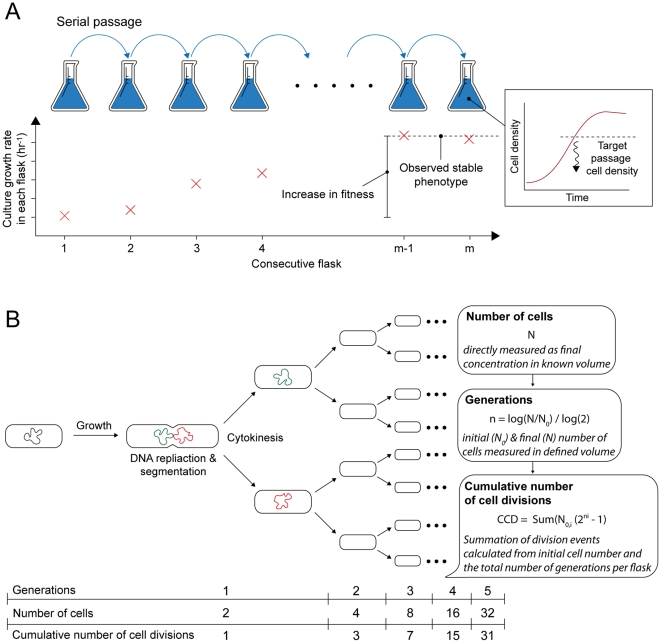
The conceptual process of ALE and calculations used to characterize ALE. (A) The top image demonstrates how serial passage is used to select for growth rate where cells are grown in flasks and passed below entering stationary phase to maintain exponential growth. During this evolution process, the growth rate of the population increases or maintains in consecutive flasks. ALE experiments are stopped when an observed stable phenotype is encountered and an overall increase in fitness can be calculated from the initial and final growth rates. The average dilution factor per passage is 1×10^−7^–1×10^−1^ on a volume per volume basis. (B) A diagram of how a single bacterial cell grows, replicates, and undergoes cytokinesis. From this process, the number of cells at a given point in time (N) can be measured, the number of generations (n) can be calculated by determining the initial number of cells in a culture (N_0_) and assuming exponential growth and a negligible death rate, and the CCD can be calculated by summing divisions from each flask.

Adaptive laboratory evolution (ALE) has become a valuable approach for the study of the genetic and biochemical basis for microbial adaptation under a strict selection pressure [1], [2]. With the availability of low-cost whole-genome sequencing platforms, the genetic changes that result in an advantageous phenotype during ALE can be readily determined [3], [4], [5], [6], [7], [8], [9]. ALE experiments, as presented in this study, are carried out for a sufficient time period to generate an apparently stable phenotype or when non-detectable changes are observed in the selected phenotypic trait (Fig. 1A). Long-term ALE experiments using *Escherichia coli* conducted by Lenski *et al.* [7], [10], [11], [12] have spanned >50,000-generations, while many shorter-term evolutions have been completed in 500 to 2,000 generations [8], [13], [14], [15], [16].

The time coordinate in ALE processes is normally scaled in terms of generations. However, DNA polymerase errors are significant sources of mutations and contribute to genetic diversity during cell growth and chromosomal replication [17]. As such, the likelihood that a mutation will occur is proportional to the number of cell divisions that take place during an ALE experiment. Therefore, one can use the cumulative number of cell divisions (CCD) in the history of the population being carried at a given time as a more meaningful measure of timescale in ALE experiments. This timescale can also be accelerated by a constant presence of a non-toxic level of a mutagen, as it increases the probability of a mutation occurring during a cell division. The CCD parameter can thus improve our understanding of ALE processes by incorporating the actual number of cells responsible for a phenotypic outcome and allow for a more precise analysis of phenotypic outcomes on a per cell basis (Fig. 1B).

We thus set out to study the dynamics of short-term ALE experiments by determining the CCD that is needed to converge to stable phenotypes and the effects of a mutagen on this process.

## Results

**Figure 2:**
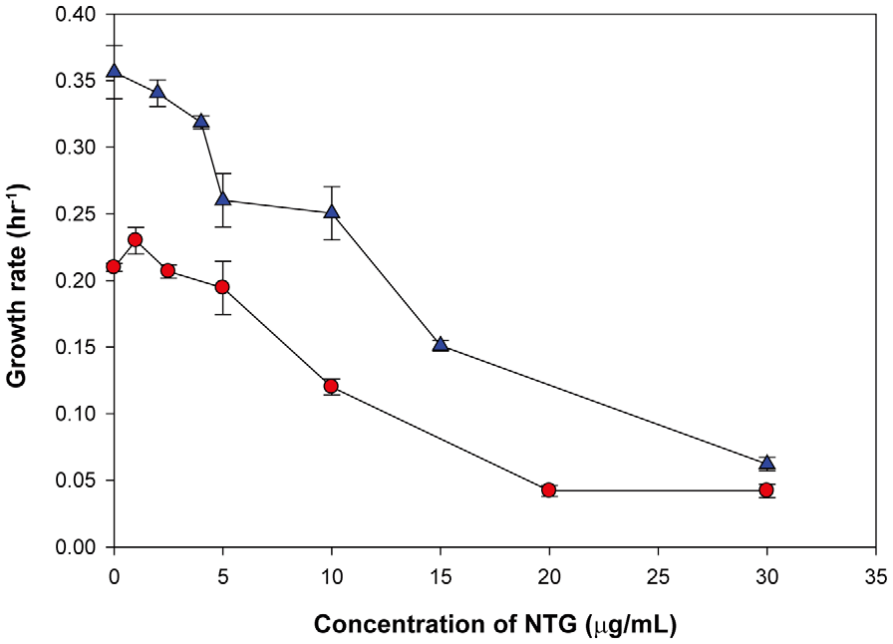
Titration of NTG on M9 minimal media supplemented with glycerol (•) or l-lactate (▴). WT *E. coli* was grown at 37°C under aerobic conditions. Growth rate was determined by measuring the OD_600_ of triplicate cultures at several time points and defined as the slope of the linear best-fit line in a plot of ln(OD_600_) versus time (hours).

Previously, we have performed a number of short-term ALE experiments using growth rate as the selection pressure. The genetic bases for the improved growth rate phenotypes were also determined using whole-genome sequencing of the endpoint strains, followed by introduction of mutations into the starting strain using allelic replacement [4], [6], [8]. Data from previously conducted experiments [6], [8], [13], [15] and additional new short-term ALE experiments with continuous exposure to a low level of a mutagen were used to compute the CCD in the population that is required to generate a reproducible phenotype. *N*-Methyl-*N*′-nitro-*N*-nitrosoguanidine (NTG) was chosen as an efficient mutagen at a level that gave excellent preservation of cell viability with continuous exposure during ALE experiments. The non-toxic amount of NTG determined by monitoring cell growth on glycerol and l-lactate minimal media was found to be 5 µg/ml and 4 µg/ml, respectively (Fig. 2). The level of NTG for ALE on glycerol minimal media was also used for *E. coli* evolution on l-1,2-propanediol (l-1,2-PDO) minimal media. A total of 24 individual ALE experiments were considered for analysis in the present study (Table 1).

**Table 1:** Adaptive laboratory evolutions used in this study.

Evolution		Replicates	GR (h^−1^)	No. of mutations	Generations	CCD	Ref.
Without NTG	Glycerol	5 (GA, GB, GC, GD, GE)	0.64±0.04	2–3	595±18	3.9×10^11^±0.5×10^11^	[4]
	l-Lactate	6 (LF, LG, LH, LI, LJ, LK)	0.54±0.04	3–8	643±75	3.8×10^11^±0.1×10^11^	[6]
	l-1,2-PDO	3 (PA, PB, PC)	0.35±0.04	6 (PA)	546±21	3.9×10^11^±0.3×10^11^	[8]
	Glucose	3 (ECOM31, ECOM32, ECOM33)	0.43±0.01	N/A	648±10	2.4×10^11^±0.2×10^11^	[15]
With NTG	Glycerol	2 (GM1, GM2)	0.74	517 (GM1)	252±12	0.93×10^11^±0.01×10^11^	This study
	l-Lactate	2 (LM1, LM2)	0.62	167 (LM1)	447±37	2.2×10^11^±0.9×10^11^	This study
	l-1,2-PDO	3 (PM1, PM2, PM3)	0.64±0.01	54–152	335±85	0.51×10^11^±0.01×10^11^	This study

GR, average stable growth rate for each condition, the endpoint culture and previous two recorded growth rates from each parallel evolution were considered in the average; N/A, not available; No. of mutations were determined by whole genome-sequencing; Generations, averaged cumulative number of generations of individual populations required for a stable phenotype to be reached; CCD, averaged cumulative number of cell divisions of individual evolved populations required for a stable phenotype to be achieved. The standard deviation of GRs for glycerol- and l-lactate-evolved populations with NTG was less than 1% of the mean values.

### Phenotypic properties

**Figure 3:**
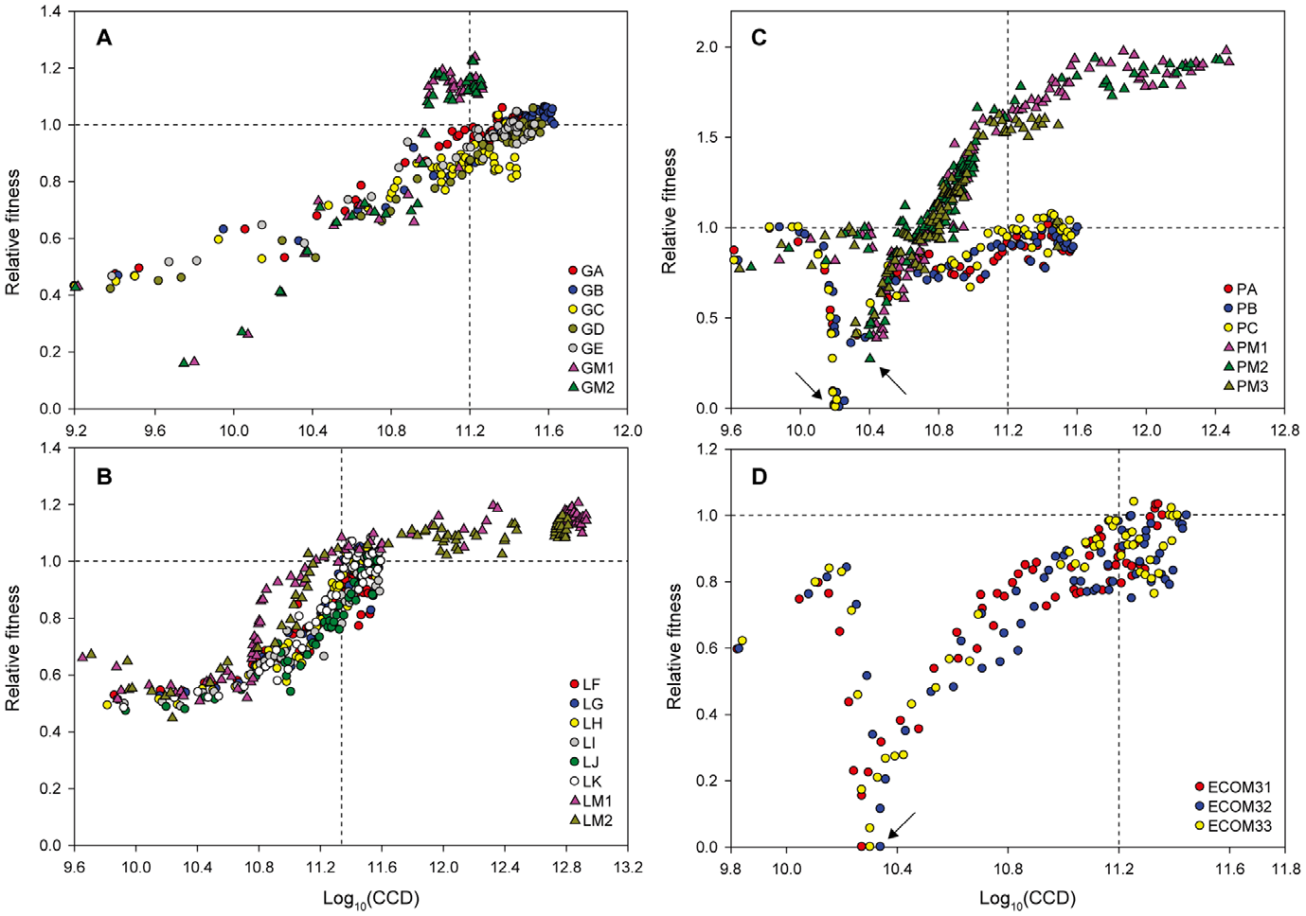
Replicate ALE of WT *E. coli* strains during adaptation to the three-carbon compounds (glycerol, l-lactate, and l-1,2-PDO) and glucose. The relative growth rate is used as a fitness criteria; i.e., growth rates are normalized to the final observed growth rates of the endpoint strains evolved without the mutagen NTG under a given selection condition. Outliers were excluded using the plotting software SigmaPlot after setting an upper and lower outlier boundary for each data set. (A) Evolution of WT *E. coli* on glycerol. Replicate ALEs were conducted without NTG (GA, GB, GC, GD, GE) [13] and with 5 µg/ml NTG (GM1, GM2). (B) Evolution of WT *E. coli* on l-lactate. l-Lactate-evolved strain without NTG (LF, LG, LH, LI, LJ, LK) [13] or with NTG (LM1, LM2). (C) Evolution of WT *E. coli* on l-1,2-PDO. The l-1,2-PDO-evolved *E. coli* strains of PA, PB, and PC were generated previously through ALE (PA, PB, PC) [8]. In this study, we have generated the l-1,2-PDO-evovled *E. coli* (PM1, PM2, PM3) under continuous exposure to NTG during ALE. The arrows indicate cells growing solely on l-1,2-PDO and no glycerol was added to support growth. (D) Evolution of ECOM3 strains on glucose. Cytochrome oxidases-deficient *E. coli* mutants were previously reported to produce d-lactic acid from glucose under aerobic conditions [15]. Three replicate ALEs (denoted by ECOM31, ECOM32, and ECOM33) were conducted to adapt the parental ECOM3 strain to growth on M9 minimal medium with glucose as the sole carbon source. The arrow indicates cells growing solely on glucose and no amino acid supplement was added to support growth.

ALE of wild-type (WT) *E. coli* K-12 MG1655 on the three-carbon compounds, glycerol, l-lactate, and l-1,2-PDO, has been conducted without and with a mutagen (Fig. 3). The main findings from these studies were:

- Glycerol-evolved *E. coli* endpoint strains (named GA, GB, GC, GD, GE) [13] reached the maximum growth rate (0.64±0.04 h^−1^) after 10^11.2^ total cell divisions. However, the endpoint *E. coli* strains evolved in the presence of NTG (GM1, GM2) underwent 10^10.9^ total cell divisions to reach the maximum growth rate of glycerol-evolved *E. coli* without NTG and had 1.2-fold increase in maximum growth rate (0.74 h^−1^) at the end point of evolution (Fig. 3A).
- l-Lactate-evolved *E. coli* strains without NTG (named LF, LG, LH, LI, LJ, LK) [6] and with NTG (LM1, LM2) exhibited 10^11.3^ and 10^11.2^ total cell divisions, respectively, and reached steady growth rates similar to the ALE experiments on glycerol (Fig. 3B). Growth rates of the evolved strains at the endpoint of evolution without and with NTG of 0.54±0.04 h^−1^ and 0.62 h^−1^ were achieved, respectively.
- l-1,2-PDO-evolved *E. coli* strains were generated through ALE (named PA, PB, PC) [8]. In a previous study, these three populations were designated eBOP12, eBOP13, and eBOP14, respectively [8]. As shown in Fig. 3C, l-1,2-PDO-evolved *E. coli* strains with NTG (PM1, PM2, PM3) showed remarkable increase in relative fitness (1.8-fold, 0.64±0.01 h^−1^) at the end of adaptive evolution over the strains evolved without NTG (0.35±0.04 h^−1^) and their CCDs required for maximum growth rate are 10^11.2^ (PA, PB, PC) and 10^10.7^ (PM1, PM2, PM3).

In addition to ALE on three-carbon substrates, ALE of multiple gene knockout strains of *E. coli* has been carried out on glucose minimal medium [14], [15], [18], [19], [20]. One such strain was a cytochrome oxidase-deficient *E. coli* mutant which produced d-lactic acid from glucose under aerobic conditions, resulting in the ECOM3 family of strains, i.e., replicate ALE endpoint populations (ECOM31, ECOM32, ECOM33) [15]. The CCD was determined for the ALE at the point where an observed stable growth rate was reached. Consistent with the above results, the ECOM3 strains showed a total of 10^11.2^ cell divisions to reach the maximum growth rate (0.43±0.01 h^−1^) during ALE (Fig. 3D).

### Genotypic properties

The genetic variations that are occurred during ALE can be readily identified by next-generation sequencing technologies. For evolved endpoint strains with NTG, we used Illumina-based sequencing to determine the mutations using the same methods as in the previous studies [4], [6], [8]. The presence of NTG markedly increased the number of mutations. A glycerol- (GM1), a l-lactate- (LM1), and multiple l-1,2-PDO-evolved strains (PM1, PM2, PM3) with NTG had 517, 167, 54, 71, and 152 mutations, respectively. The single nucleotide differences between the evolved strains with NTG and the parental WT *E. coli* reference strain are described in Table S1. A comparison of the mutations found in the cells evolved with and without NTG will now be presented to characterize the impact of the mutagen.

**Figure 4:**
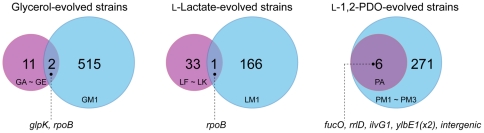
Venn diagram of mutations shared between the evolved strains with and without NTG. Surface areas are not proportional to members contained in each set. Light blue and purple circle represents the mutations found in total number of strains evolved with and without NTG, respectively. The single nucleotide differences between the evolved strains with NTG and the parental WT *E. coli* reference strain are described in Table S1. Whole-genome sequencing of ALE endpoint strains evolved on glycerol, l-lactate, and l-1,2-PDO without NTG has been previously reported [4], [6], [8]. The *ylbE1* gene of l-1,2-PDO-evolved *E. coli* without NTG had two mutations [8].

- In the GM1 strain, a total of 459 mutations among 517 discovered mutations were found within the coding region. Although most SNPs resulted in an amino acid change, 75 SNPs were synonymous mutations. In addition, 58 and 40 mutations were found in intergenic regions and genes annotated only as conserved or predicted proteins (i.e., unknown function genes), respectively. Unlike glycerol-evolved strains without NTG (GA, GB, GC, GD, GE), many SNPs were identified in genes of glycerol metabolism from the GM1 strain, namely; *glpD* (aerobic glycerol 3-phosphate dehydrogenase), *glpT* (glycerol 3-phosphate MFS transporter), *ugpA/B* (glycerol 3-phosphate ABC transporter), *dhaR* (dihydroxyacetone regulator), *gpmM* (phosphoglycerate mutase), and *acs* (acetyl-CoA synthetase). These mutations found might confer the GM1 advantageous growth phenotype on glycerol minimal media. The number of mutations found in glycerol-adapted strains without NTG was between two and three [4]. Mutations in the non-mutagenized strains were found in genes encoding the two major subunits of RNA polymerase (*rpoB* and *rpoC*), which are conferring the largest change in growth rate. In addition, all sequenced clones had mutations in the *glpK* gene coding for glycerol kinase, which catalyzes the first step in glycerol catabolism [4]. These mutations (*glpK* and *rpoB*) were also detected in the adapted strain with NTG, GM1 (Fig. 4).
- In the LM1 strain, a total of 167 mutations were detected from whole-genome sequencing. Among them, 137 and 17 mutations were discovered in coding regions of annotated genes and genes annotated only as conserved or predicted proteins, respectively. Whole-genome sequencing of the LM1 showed many mutations in relevant central metabolic pathways that were not previously identified in l-lactate-evolved strains without NTG (LF, LG, LH, LI, LJ, LK); *aldA* (aldehyde dehydrogenase A), *gapA* (glyceraldehyde 3-phosphate dehydrogenase), *livH/J* (branched amino acids ABC transporter), *acs* (acetyl-CoA synthetase), and *ydjG* (methylglyoxal reductase). Like *E. coli* strains evolved without mutagen on l-lactate, the LM1 had a mutation in the *rpoB* (RNA polymerase β subunit) gene (Fig. 4), while conversely, there was no mutation in *lldD* (l-lactate dehydrogenase), which catalyzes the first step in l-lactate catabolism in *E. coli*. Accounting for SNPs, deletions, and insertions, we found a total of 34 mutations across six l-lactate-evolved strains evolved with no mutagen [6]. Those mutations affected many different genes with a broad range of cellular functions, but the majority of mutations belong to genes with primary functions relating to metabolism, regulation, or the cell envelope. The most frequently mutated metabolic gene was *rph*-*pyrE*, which is involved in pyrimidine biosynthesis [6]. However, this mutation was not found in l-lactate-evolved strains with mutagen.
- In the PM1, PM2, and PM3 strains, a total of 277 mutations corresponding to 217 unique genomic positions were detected across all three strains from whole-genome sequencing. Of these mutations, 53 were detected in intergenic regions at 45 unique positions in the genome across all three strains. The evolved strains on l-1,2-PDO with NTG had a mutation in a regulatory gene (*rpoD*; sigma 70 factor or *cyaA*) or the RNA polymerase (*rpoB* or *rpoC*; RNA polymerase β' subunit) which were not reported previously. However, the beneficial effects of these mutations on adaptation to other three carbon substrates (glycerol and l-lactate) are well known [4], [6]. Interestingly, all mutations found in a l-1,2-PDO-evovled strain without NTG (PA) were also detected in l-1,2-PDO-adapted strains with NTG (Fig. 4). A total of six mutations were found to have accumulated in the l-1,2-PDO-adapted strain (PA) [8]. Five of the six mutations were in coding regions, and there was an IS*5* insertion in the region between the *fucAO* and *fucPIKUR* operons, which had caused constitutive activation of the *fucAO* operon [8]. Also, a SNP was also found in the *fucO* gene encoding the l-1,2-PDO oxidoreductase in the PA strain, which catalyzes the first step of l-1,2-PDO catabolism in *E. coli*.

## Discussion

ALE is increasingly being used to study the dynamics of bacterial adaptation, its underlying genetic basis, and to identify the altered biochemical mechanisms [21]. The measure of time for these experiments should be scaled in terms of the probability of generating mutations and the number of cells needed to select for them through competition. In this study, we found: 1) that the CCD needed for the generation of reproducible growth phenotypes during short-term ALE is about 10^11.2^, 2) that this rate can be accelerated using a continuous exposure to a nontoxic low level of a mutagen, and 3) that in the presence of the mutagen, the number of mutations that are explored by whole-genome sequencing increases significantly, leading to improved growth phenotypes as compared to the endpoint strains generated without the mutagen.

The main advance of the present study is in calculating the CCD that was required for *E. coli* cells to show the reproducible phenotype during short-term ALE. Regardless of carbon sources (three-carbon compounds and glucose) and strain backgrounds (WT and knockout *E. coli*), the CCD were approximately 10^11.2^ to generate the observed stable growth phenotype (Fig. 3). The CCD has been used as a unit of time to estimate bacterial mutation rate [22], [23]. Luria and Delbrück [22] devised the fluctuation test, assuming that the mutation is proportional to the number of cells present at that time and that the number of cell divisions is approximately equal to the number of cells in the population (because the cell population is so large (>10^7^)). If we use the cumulative generations as the timescale of ALE, the mutation rate will be overestimated because the cumulative generations cannot reflect the real number of cells in a culture at a given time. The CCD incorporates the actual number of cells involved in an evolution experiment specifically into a parameter that can be correlated to a phenotypic outcome in an experiment. Thus, usage of the CCD allows for a more precise analysis of phenotypic outcomes on a per cell basis. Furthermore, the use of the CCD accounts for variability and allows for a more precise comparison of multiple evolution experiments because the number of cells passed serially from one flask to the next can vary across a range, as we presented here.

Cells copy their genetic material with exceptional accuracy (the spontaneous mutation frequency in *E. coli* can be as low as 4×10^−10^ base substitution mutations per base pair per generation) [24]. The robust amplification of the effects of an individual molecular event resulting from such accuracy makes it difficult to study the mutations. Mutagens can speed up the rate of these spontaneous mutations during ALE experiments. The continuous exposure of *E. coli* to non-toxic levels of NTG during ALE decreases the CCD required for a maximum observed stable growth rate. In addition, NTG treatment has been shown to generate a superior growth phenotype at the endpoint of ALE as compared to ALE without NTG. Comparisons of whole-genome sequences between the non-mutagenized evolved *E. coli* strain and descendants exposed to NTG mutagenesis revealed 257 mutations per genome on average, while spontaneously evolved *E. coli* strains showed between 2 to 8 mutations. Clearly, it is impractical to investigate all of these mutations by allelic replacement. However, with the increasing prevalence and decreasing cost of genome resequencing, along with the emergence of technologies such as MAGE [25] to rapidly and accurately introduce mutations into a genome so that causality can be determined, we envision that the ability to evaluate causality with a relatively large number of mutations will be possible. Furthermore, in our experience, because only very few mutations of ALE without mutagen prove dominant, it is feasible to determine the genetic basis for adaptation [4], [6], [8].

Given the three different evolution conditions, a high-level comparison can be made about the path to higher fitness taking in account the overlap of genes in which mutations were found with and without mutagen. For increased fitness in l-1,2-PDO strains, the evolutionary trajectory is rather restricted as all of the mutations found in the no mutagen evolution were also in the strains evolved with mutagen. For the glycerol evolution, the path to increased fitness is broader as the overlap is only a few genes. These two overlapping mutated genes (*glpK* and *rpoB*), in particular, solely accounted for most of the increase in fitness observed for evolution on glycerol, thus proving to be important in the glycerol evolution [4]. Lastly, for the l-lactate evolution, the path to higher fitness seems rather diverse given a large amount of mutations found in multiple evolutions without mutagen and an overlap of only 1 gene, with the resequenced evolution with a mutagen, *rpoB*. With further experimentation in terms of replicates and under different conditions, the path to higher fitness and flexibility in which genes mutate as a result will become clearer.

Taken together, this information can be used to define studies examining the kinetics of the ALE process and further the possibility of developing mathematical descriptions of the dynamics of the selection process that takes place during ALE. With the availability of inexpensive whole-genome sequencing, such dynamic models can be generated with a full genetic basis. In addition, this information will not only aid in understanding adaptation, but can be leveraged to engineer and design desirable microbial stains.

## Materials and Methods

### Strains and media

A WT *E. coli* K-12 MG1655 strain was used as a parent strain for adaptive evolution on glycerol, l-lactate, and l-1,2-PDO with NTG (Catalogue number 05343; Sigma Aldrich). Evolutions were carried out at 37°C using 200 ml of M9 minimal medium supplemented with 2 g/liter of each carbon source in 500-ml Erlenmeyer flasks containing magnetic stir bars for aeration. M9 minimal medium contained (per liter of deionized water) 0.8 g of NH_4_Cl, 0.5 g of NaCl, 7.5 g of Na_2_HPO_4_·2H_2_O, and 3.0 g of KH_2_PO_4_. The following components were sterilized separately and then added (per liter [final volume] of medium): 2 ml of 1 M MgSO_4_, 0.1 ml of 1 M CaCl_2_, and 0.5 ml of a trace element solution containing (per liter) 1 g of FeCl_3_·6H_2_O, 0.18 g of ZnSO_4_·7H_2_O, 0.12 g of CuCl_2_·2H_2_O, 0.12 g of MnSO_4_·H_2_O, and 0.18 g of CoCl_2_·6H_2_O. During the early stage of adaptive evolution on l-1,2-PDO, the minimal medium was also supplemented with 2 g/liter of glycerol and the concentration of these compounds was gradually decreased while the l-1,2-PDO concentration was increased to keep the total carbon source concentration in the minimal medium 2 g/liter.

### ALE with NTG

At the start of adaptive evolution, the WT strain was cultured on solid M9 minimal medium containing 2 g/liter of carbon source and incubated overnight at 37°C. A single colony was selected from the plate that was incubated, resuspended in 10 µl of sterile water, and inoculated into two or three 500-ml Erlenmeyer flasks containing 200 ml of M9 minimal medium supplemented with 2 g/liter of appropriate substrate. NTG was added as an efficient mutagen at a level that gave excellent preservation of viability with continuous exposure. The flasks were incubated at 37°C using a stir bar for mixing and aeration (∼1,000 rpm). For adaptive evolution cultures, the optical density at 600 nm (OD_600_) was determined and cells were transferred into fresh medium. The dilution factor used for each passage was adjusted daily to account for changes in the growth rate (typically between 2.5×10^4^ and 1.2×10^6^ cells were transferred during each inoculation) and to ensure that cultures did not enter the stationary phase before the next passage. ALE experiments were ended when no significant change in the culture growth rate was observed over several passages (typically, the growth rates of the previous ten flasks were considered). Replicate cultures were evolved concurrently under identical conditions. Cultures were screened every other day for contamination by performing PCR with primers for the V2 region of 16S rDNA genes and Sanger sequencing [8]. Samples were stored at −80°C every day over the course of evolution.

### Titration of NTG to a nontoxic level

To determine the non-toxic level of NTG to *E. coli* K-12 MG1655 cell growth, various amounts of NTG were tested. Growth rate was determined by measuring the OD_600_ of triplicate cultures at several time points at which the OD_600_ was >0.05 but <0.3. The growth conditions used were identical to the conditions used for ALE, except that flasks were placed in a 37°C water bath instead of the 37°C air incubator used for ALE. The growth rate was defined as the slope of the linear best-fit line in a plot of ln(OD_600_) versus time (hours).

### Calculation of CCD

The CCD for each replicate evolution was calculated using the equation:
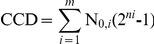
Where, *N_0,i_* is the initial number of cells in each flask during the evolution, *n* is the number of generations for each flask, and *m* is the total number of individual flask cultures used in the serial ALE process. The number of generations for each flask, *n*, was calculated using the equation:
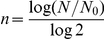
Where, *N* is the final number of cells in a flask at the time of passage to the next flask (see Fig. 1B). The initial and final numbers of cells were estimated daily by measuring the OD_600_ using a Biomate 3 spectrophotometer (Thermo Scientific) and determining how many cells were in 1 L of M9 minimal medium at a normalized OD_600_ of 1. A value of 7.87×10^10^ cells · L^−1^ · OD_600_^−1^ and 2.32×10^10^ cells· L^−1^ · OD_600_^−1^ was used calculate cell numbers for evolved populations without and with the NTG, respectively. The CCD calculation assumes that each cell is viable and the death rate is negligible, the cells are growing exponentially throughout the ALE experiment, and the cells are dividing by binary fission.

### Whole-genome sequencing

Five micrograms of genomic DNA isolated from a single clone of the endpoint glycerol-, l-lactate, and l-1,2-PDO-evolved populations with continuous exposure to a low level of NTG was used to generate a genomic DNA library using an Illumina genomic DNA library generation kit by following the manufacturer's protocol (Illumina Inc., San Diego, CA). Briefly, bacterial genomic DNA was fragmented by nebulization. The ends of fragmented DNA were repaired by T4 DNA polymerase, Klenow DNA polymerase, and T4 phosphonucleotide kinase. The exonuclease-negative Klenow DNA polymerase was then used to add an A base to the 3′ end of the DNA fragments. After ligation of the adapters to the ends of the DNA fragments, the ligated DNA fragments were subjected to electrophoresis on a 6% 1× Tris-borate-EDTA (TBE) gel. DNA fragments ranging from 190 bp to 220 bp long were recovered from the gel and purified using a Qiagen minigel purification kit. Finally, the adapter-modified DNA fragments were enriched by PCR. The final concentration of the genomic DNA library was determined by using a NanoDrop instrument (Thermo Scientific), and the results were validated by using a 6% 1× TBE gel. The genomic DNA library was used to generate a cluster on a Flowcell by following the manufacturer's protocol. The V2 genomic sequencing primer was used for all DNA sequencing. A 36-cycle sequencing program was used with an Illumina genome analyzer II by following the manufacturer's protocol.

### Genome sequence assembly and identification of polymorphism

The Illumina output for the resequencing run was first curated to remove any sequences containing a period. We then used MosaikAligner, developed by M. P. Stromberg and G. T. Marth (unpublished data), to iteratively align reads with the *E. coli* reference sequence (gi 48994873); for each iteration a limit was placed on the number of alignment mismatches allowed. This iterative limit increased from 0 to 5, and unaligned reads were used as input for the next iteration, which had a more lenient mismatch limit. An in-house script (available upon request) was then used to compile the read alignments into a nucleotide resolution alignment profile. The consistency and coverage were then assessed to identify likely polymorphic locations. Locations at which the count for a single-nucleotide polymorphism (SNP) was greater than twice the count for the nucleotide matching the reference sequence were considered to likely be polymorphic locations. False-negative rates were determined by this sequencing method by carrying out polymorphism identification analysis using an *E. coli* reference sequence which had 1,000 SNPs, deletions, and insertions added at random and known locations. Mutations were not permitted to overlap. The rate of detection of SNPs was determined by calculating the fraction of each type of mutation that was marked as polymorphic by the script described above when sequence data from an end point were mapped on the mutated reference genome.

## Supporting Information

Table S1 **Table showing the single nucleotide differences between the evolved strains with NTG (GM1, LM1, PM1, PM2, and PM3) and the parental WT** ***E. coli*** **reference strain.** GM1, the endpoint glycerol-evolved *E. coli* strain with NTG; LM1, the endpoint l-lactate-evolved *E. coli* strain with NTG; PM1∼PM3, the endpoint l-1,2-PDO-evolved *E. coli* strains with NTG; Position in reference, genomic position in wild-type *E. coli* K-12 MG1655; AA, amino acid.
(XLSX)

## References

[1] Lenski RE, Mongold JA, Sniegowski PD, Travisano M, Vasi F (1998). Evolution of competitive fitness in experimental populations of *E. coli*: What makes one genotype a better competitor than another?. Antonie Leeuwenhoek.

[2] Elena SF, Lenski RE (2003). Evolution experiments with microorganisms: the dynamics and genetic bases of adaptation.. Nat Rev Genet.

[3] Shendure J, Porreca GJ, Reppas NB, Lin X, McCutcheon JP (2005). Accurate multiplex polony sequencing of an evolved bacterial genome.. Science.

[4] Herring CD, Raghunathan A, Honisch C, Patel T, Applebee MK (2006). Comparative genome sequencing of *Escherichia coli* allows observation of bacterial evolution on a laboratory timescale.. Nat Genet.

[5] Velicer GJ, Raddatz G, Keller H, Deiss S, Lanz C (2006). Comprehensive mutation identification in an evolved bacterial cooperator and its cheating ancestor.. Proc Natl Acad Sci USA.

[6] Conrad T, Joyce A, Applebee MK, Barrett C, Xie B (2009). Whole-genome resequencing of *Escherichia coli* K-12 MG1655 undergoing short-term laboratory evolution in lactate minimal media reveals flexible selection of adaptive mutations.. Genome Biol.

[7] Barrick JE, Yu DS, Yoon SH, Jeong H, Oh TK (2009). Genome evolution and adaptation in a long-term experiment with *Escherichia coli*.. Nature.

[8] Lee D-H, Palsson BO (2010). Adaptive evolution of *Escherichia coli* K-12 MG1655 on a non-native carbon source, l-1,2-propanediol.. Appl Environ Microbiol.

[9] Atsumi S, Wu T-Y, Machado IMP, Huang W-C, Chen P-Y (2010). Evolution, genomic analysis, and reconstruction of isobutanol tolerance in *Escherichia coli*.. Mol Syst Biol.

[10] Travisano M, Lenski RE (1996). Long-term experimental evolution in *Escherichia coli*. IV. Targets of selection and the specificity of adaptation.. Genetics.

[11] Pelosi L, Kuhn L, Guetta D, Garin J, Geiselmann J (2006). Parallel changes in global protein profiles during long-term experimental evolution in *Escherichia coli*.. Genetics.

[12] Nadege P, Estelle C, Richard EL, Dominique S (2007). Evolution of global regulatory networks during a long-term experiment with *Escherichia coli*.. BioEssays.

[13] Fong SS, Joyce AR, Palsson BO (2005). Parallel adaptive evolution cultures of *Escherichia coli* lead to convergent growth phenotypes with different gene expression states.. Genome Res.

[14] Hua Q, Joyce AR, Fong SS, Palsson BO (2006). Metabolic analysis of adaptive evolution for *in silico*-designed lactate-producing strains.. Biotechnol Bioeng.

[15] Portnoy VA, Herrgard MJ, Palsson BO (2008). Aerobic fermentation of d-glucose by an evolved cytochrome oxidase-deficient *Escherichia coli* strain.. Appl Environ Microbiol.

[16] Stanek M, Cooper T, Lenski RE (2009). Identification and dynamics of a beneficial mutation in a long-term evolution experiment with *Escherichia coli*.. BMC Evol Biol.

[17] Camps M, Naukkarinen J, Johnson BP, Loeb LA (2003). Targeted gene evolution in *Escherichia coli* using a highly error-prone DNA polymerase I.. Proc Natl Acad Sci USA.

[18] Fong SS, Palsson BO (2004). Metabolic gene-deletion strains of *Escherichia coli* evolve to computationally predicted growth phenotypes.. Nat Genet.

[19] Fong SS, Nanchen A, Palsson BO, Sauer U (2006). Latent pathway activation and increased pathway capacity enable *Escherichia coli* adaptation to loss of key metabolic enzymes.. J Biol Chem.

[20] Feist AM (2008). Model-driven metabolic engineering of *Escherichia coli*: A systems biology approach.

[21] Palsson BO (2011). Adaptive laboratory evolution.. Microbe.

[22] Luria SE, Delbruck M (1943). Mutations of bacteria from virus sensitivity to virus resistance.. Genetics.

[23] Galán J-C, Turrientes M-C, Baquero M-R, Rodríguez-Alcayna M, Martínez-Amado J (2007). Mutation rate is reduced by increased dosage of *mutL* gene in *Escherichia coli* K-12.. FEMS Microbiol Lett.

[24] Parkhomchuk D, Amstislavskiy V, Soldatov A, Ogryzko V (2009). Use of high throughput sequencing to observe genome dynamics at a single cell level.. Proc Natl Acad Sci USA.

[25] Wang HH, Isaacs FJ, Carr PA, Sun ZZ, Xu G (2009). Programming cells by multiplex genome engineering and accelerated evolution.. Nature.

